# Potential therapeutic effects of GS-441524 and GC376 in cats with feline infectious peritonitis

**DOI:** 10.18849/ve.v7i1.522

**Published:** 2022-02-02

**Authors:** Omid Nekouei+, Sophie St-Hilaire+, Pak Chun Hui+, Karen Chan+, Isabel Sumyi Chan+, Sum Yuet Lorraine Ngan+, Yion Chan+, Ka Po Chung+, Sunguk Hong+, Hiu Man Chan+, Hoi Lam Iris Or+, Fong Yuen Chan+, Hei Tung Yim+, Vanessa R. Barrs+

**Keywords:** STRESS

## Abstract

PICO question

In cats with feline infectious peritonitis (FIP), does treatment with the nucleoside analogue GS-441524 or the protease inhibitor GC376, compared to supportive measures alone, lead to longer survival times?

Clinical bottom line

Category of research question

Treatment

The number and type of study designs reviewed

Five studies, including four uncontrolled interventional studies and one case-series were critically reviewed

Strength of evidence

Moderate

Outcomes reported

The reviewed studies collectively provide moderate evidence in support of the application of GS-441524 or GC376 to extend the survival time of cats suffering from feline infectious peritonitis

Conclusion

While these antiviral drugs are considered the most likely options for FIP treatment, more robust evidence should be obtained through well-designed randomised controlled trials to verify the observed positive effects in treating various forms of the disease and the potential long-term side effects. However, the ethical dilemmas of conducting double blinded placebo-controlled trials, which by necessity include untreated cats with an invariably fatal disease are recognised

## How to apply this evidence in practice

The application of evidence into practice should take into account multiple factors, not limited to: individual clinical expertise, patient’s circumstances and owners’ values, country, location or clinic where you work, the individual case in front of you, the availability of therapies and resources.

Knowledge Summaries are a resource to help reinforce or inform decision making. They do not override the responsibility or judgement of the practitioner to do what is best for the animal in their care.

## Clinical scenario

A 14 month old male domestic short-haired cat is presented to you with progressive signs of lethargy, inappetence, jaundice, fluctuating fever, and distended abdomen. Your physical examination and complete blood work point to the possibility of feline infectious peritonitis (FIP). Abdominal effusion sample is a non-septic exudate with high protein and low cellularity. A molecular diagnostic test (reverse transcription quantative polymerase chain reaction [RT-qPCR]) detects high levels of feline coronavirus in the effusion sample and immunocytochemistry of the effusion confirms the diagnosis. A colleague suggests using GS-441524 or GC376 to treat the infected cat to increase his quality of life. However, you are not sure about the clinical efficacy and safety of these antiviral drugs due to the limited evidence available.

## The evidence

Following the eligibility criteria, five studies were deemed relevant to the PICO question, including a case-series (Dickinson et al., 2020) and four interventional studies (Kim et al., 2016; Murphy et al., 2018; Pedersen et al., 2018; and Pedersen et al., 2019). The case-series was limited to only four cases with neurological FIP treated with different regimens of GS-441524, providing weak evidence for the treatment efficacy due to the nature of the study. Although all of the four experimental studies showed a range of positive clinical effects from the treatment with either GS-441524 (Murphy et al., 2018; and Pedersen et al., 2019) or GC376 (Kim et al., 2016; and Pedersen et al., 2018), none of them included an independent control group. Therefore, all four studies were prone to different types of bias. Although there were similarities among the treatments in these studies, they were not directly comparable due to differences in the clinical presentation of cases and the details of interventions. It has been shown that nearly all cats with clinical FIP die within a few weeks to a few months of diagnosis with supportive measures alone. Overall, our assessment provides moderate support to these treatments to increase the survival times of affected cats.

## Summary of the evidence

**Dickinson et al. (2020):** 

Population:	Four cats, 7–18 months old, with naturally occurring neurological and ocular feline infectious peritonitis (FIP).
Sample size:	Four cats.
Intervention details:	FIP diagnosis was presumed in the cats based on the combination of patient signalment and clinical findings as well as supportive laboratory findings including hyperglobulinameia, low albumin/globulin ratios, feline coronavirus antibody titres and response to the anti-viral treatment. All cats were treated with 5 mg/kg GS-441524, subcutaneously (SC), once daily as follows: Two cats were treated for 14 weeks (Cases 1 and 2). Case 3 was first treated for 15 weeks. Within 36 hours after cessation of the initial treatment, clinical signs recurred; therefore, the cat went under the second round of treatment for 12 weeks. Case 4 with severe signs was treated for a total of 19 weeks in three courses: 1) initial 4 weeks with 5 mg/kg (along with prednisolone acetate 1% and dorzolamide 2% eye drops for the first 3 weeks); 2) with an increased dosage of 8 mg/kg for 10 more weeks; 3) with an increased dosage of 10 mg/kg for 5 additional weeks.
Study design:	Case report (case-series).
Outcome studied:	Resolution of clinical signs (through physical, behavioural neurological, and ophthalmic examinations). In Case 4, normalisation of magnetic resonance imaging (MRI) and cerebrospinal fluid (CSF) findings and resolution of cranial and caudal ocular segment disease with ocular imaging. Number of days the cases lived off the treatment.
Main Findings (relevant to PICO question)	Three cats (Cases 1, 2, and 4) had long-term resolution of the clinical signs and remained normal until the publication of this study (at least 1 year). A dosage of 5 mg/kg, SC, once daily for 12–14 weeks was sufficient to treat the two less severe cases (Cases 1 and 2). For Case 3, limited efficacy associated with the 5 mg/kg dose was observed and repeated treatments at the same dosage only improved the clinical signs. Injections of GS-441524 were associated with localised skin reactions and discomfort. Once the treatment was stopped, there was a rapid clinical regression. This cat was euthanised. For Case 4, increased dosage of the treatment was associated with improvements in the clinical and MRI signs, biochemical abnormalities of CSF, and weight gain. Treating neurological FIP may require a higher dosage of GS-441524 than those for non-neurological FIP.
Limitations:	Case-series (being at the bottom of the pyramid of evidence). Small sample size. The disease course and intensity was different among the cases. Treatment course varied among the cases. The study was limited only to neurological FIP cases.

**Kim et al. (2016):** 

Population:	Eight laboratory-bred specific-pathogen-free (SPF) cats (8–10 months old).
Sample size:	Eight cats.
Intervention details:	Two independent experiments were conducted: In the first experiment, four cats were intraperitoneally (IP) inoculated with a cat-passaged serotype I FIPV. Following infection, they developed lymphopenia and clinical signs, including inapparent or mild ascites, 14–20 days post- In the second experiment, another four cats were infected in the exact same manner, but their ascites was allowed to progress to more profound, classical abdominal effusions resembling cats with naturally occurring feline infectious peritonitis (FIP). FIP diagnosis was made based on the develpment of supportive signs and laboratory abnormalities within 2–3 weeks of experimental inoculation, including high fever, weight loss, jaundice and absolute fever. Ascites was also presented in 6/8 cats. Both groups were treated with GC376 at 5–10 mg/kg, twice daily, subcutaneously, when they developed absolute lymphopenia and typical clinical signs. Cats were treated for 14–20 days, except for two cats that were euthanised after 4 and 7 days following the antiviral treatment.
Study design:	Experimental study (uncontrolled).
Outcome studied:	Rectal. Body weight. Lymphocyte count over. Viral load in the macrophages from ascites (in two cats).
Main Findings (relevant to PICO question)	Treatment with GC376 led to the reversal of the disease progression when initiated at the advanced clinical stages of FIP in six cats (four from the first and two from the second experiment). 2/8 cats were euthanised due to the severity of their clinical signs. The six recovered cats remained healthy with no signs of relapse during 8 months of the follow-up period.
Limitations:	Small sample. Lack of independent control group. Limited generalisability due to using lab-bred cats and generating experimental infection (IP inoculation of a cat-passaged serotype I FIPV) that could be different from the natural infection.

**Murphy et al. (2018):** 

Population:	12 laboratory-bred specifc-pathogen-free (SPF) cats (6–9 months old).
Sample size:	12 cats.
Intervention details:	Cats were intraperitoneally (IP) inoculated with the cat-passaged serotype I FIPV. Feline infectious peritonitis (FIP) diagnosis was made based on the development of supportive signs and laboratory abnormalities within 2–3 weeks of experimental inoculation, including fever, inappetence, lethargy, weight loss, ascites, hyperbilirubinaemia, and absolute lymphopenia. 10/12 cats demonstrated clinical signs of wet FIP within 10–18. Two cats that did not develop disease signs served as controls (only) for normal blood lymphocyte counts and rectal. Infected cats with clear clinical signs of FIP were divided into two groups of five, A & B, and treated with GS-441524, once daily, for 2 weeks, with 5 mg/kg and 2 mg/kg doses, respectively. Cats with recurrent disease signs were treated with the same regimen for 2 additional. There were no ‘untreated’ control groups due to ethical reasons (published data on the fatal outcome of similar experiments). The post-treatment observation period was 8 months.
Study design:	Experimental study (uncontrolled).
Outcome studied:	Rectal temperature. Blood lymphocyte counts. Appetite. Bilirubin levels. Presence of ascites.
Main Findings (relevant to PICO question)	2 weeks of GS-441524 treatment at a dosage of 2 or 5 mg/kg subcutaneously q24h rapidly reversed the clinical signs and prevented FIP-associated mortality in the study cats. 2/10 treated cats had recurrences of disease at 4 and 6 weeks post treatment. A second 2 week course of GS-441524 treatment resulted in rapid resolution of disease All 10 treated cats remained normal for, at least, the 8 months of follow-up.
Limitations:	Small sample. Lack of independent control. Limited generalisability due to using lab-bred cats and generating experimental infection (intraperitoneal inoculation of a cat-passaged serotype I FIPV) that could be different from the natural infection. Conflict of interest: three of the authors are employees of the manufacturer of the drug and hold stock interests in the company.

**Pedersen et al. (2018):** 

Population:	20 client-owned cats, 3.3–82 months old, presented with various forms of naturally occurring feline infectious peritonitis (FIP) to a clinic (at University of California, Davis, USA).
Sample size:	20 cats.
Intervention details:	FIP diagnosis was presumed in the subjects upon entry based on signalment, clinical history, examination of prior laboratory test results, physical examination, and repeating basic blood and effusion analyses. The presence of FIPV was further confirmed by quantitative reverse transcription polymerase chain reaction (qRT-PCR) either from abdominal or thoracic effusions taken at the time of admission (or at necropsy). GC376 was administered subcutaneously at 15 mg/kg q12h, initially for 2 weeks. The courses of treatment were progressively extended as long as the cats remained responsive to GC376. Cats were discharged to their owners when a positive response to treatment was noted (usually within 5 days), and the owners continued the injections following appropriate instructions from veterinarians. The eventual duration and dosage of the treatment was variable among the subjects.
Study design:	Clinical trial (uncontrolled).
Outcome studied:	Remission of clinical signs (e.g., body temperature, appetite, the presence of neurological signs). Weight gain. Abdominal effusion and blood serum chemistry. The occurrence of relapse.
Main Findings (relevant to PICO question)	19/20 cats showed progressive improvement in health during the first 4 weeks of treatment. However, 13/20 cats ultimately succumbed due to the disease recurrence. Failure to achieve sustained remission (or prevent relapse) was associated with either a high incidence of neurological disease or recurrence of abdominal lesions. Attempt to treat neurological signs by increasing drug dose and treatment duration slowed the progression but did not reverse the signs. The other seven cats experienced long-term remission and were categorised as potential treatment successes (at least in 12 weeks of follow-up). Sustained remissions were more likely to occur in cats <18 weeks of age, and less likely in cats >18 weeks of age presenting with dry, dry-to-wet, or ocular disease. The main adverse effects of treatment included transient stinging upon injection (with dermal reaction) and retarded juvenile dentition development.
Limitations:	No independent control group (due to ethical concerns). Inconsistent duration of treatment for some studied cats. Injections of the drug were partially conducted by the cat owners. Cats with neurological signs were not included in the study.

**Pedersen et al. (2019):** 

Population:	31 client-owned cats, 3.4–73 months old, presented with naturally occurring feline infectious peritonitis (FIP) to a clinic (at University of California, Davis).
Sample size:	31 cats.
Intervention details:	FIP diagnosis was presumed in the subjects upon entry based on signalment, clinical history, examination of prior laboratory test results, physical examination, and repeating blood and effusion analyses. Thoracic or abdominal effusions from wet FIP cases were confirmed positive for feline coronavirus by quantitative reverse transcription polymerase chain reaction (qRT-PCR). Cats with signs of non-effusive FIP were further tested by abdominal and thoracic ultrasonography for primary lesions such as low-volume effusions, organomegaly and lymphadenopathy. GS-441524 was administered at 2 mg/kg, subcutaneously, q24h for at least 12 weeks. The initial treatment was extended for one or more weeks in cats that still had abnormal serum protein values. The dosage was increased during later stages of the trial from 2–4 mg/kg in cases with extended treatment or disease relapse.
Study design:	Clinical trial (uncontrolled).
Outcome studied:	Remission of clinical signs (e.g., body temperature, appetite, the presence of neurological signs). Weight gain. Resolution of abdominal effusion and blood serum chemistry abnormalities. The occurrence of relapse.
Main Findings (relevant to PICO question)	4/31 cats died or euthanised within the first 2–5 days of treatment due to severe disease and other complications and another cat was euthanised after 26 days due to no treatment 26/31 cats completed the planned course of treatment (12 weeks or longer). From these 26, only eight suffered relapses over the follow-up period and were retreated with the original or higher dosage (4 mg/kg) for a second or third round of treatment. Among the eight cats, only one with neurological signs failed to respond to the second round of treatment and was euthanised. Overall, 25/26 cats achieved sustained remission of FIP (i.e., long-term survivors > 9 months).
Limitations:	No independent control group (due to ethical concerns). Inconsistent courses of treatment for eight study cats. Injections of the drug were partially conducted by the cat owners. Cats with obvious neurological and ocular FIP were not included in the study (limited generalisability).

## Appraisal, application and reflection

Although GS-441524 (nucleoside analogue) and GC376 (protease inhibitor) are the most recommended antiviral drugs for treating and increasing the longevity of cats with feline infectious peritonitis (FIP) they are not commercially available (Kennedy, 2020). This Knowledge Summary was prepared to address a critical question posed by many small animal practitioners regarding the efficacy of these drugs in a clinical setting.

Following the specified eligibility criteria, five studies were found relevant to our PICO question, including a case-series (Dickinson et al., 2020) and four interventional studies (Kim et al., 2016; Murphy et al., 2018; Pedersen et al., 2018; and Pedersen et al., 2019). These studies provided different levels of support to the efficacy of GS-441524 or GC376 in resolving the clinical signs and extending the survival time of cats with FIP, especially when the treatment was administered in an earlier stage of the disease and/or continued for an extended period. Three studies investigated the application of GS-441524 (Murphy et al., 2018; Pedersen et al., 2019; and Dickinson et al., 2020;), and two studies involved GC376 (Kim et al., 2016; and Pedersen et al., 2018).

These studies generally showed the safety and positive effects of GC376 or GS-441524 as evidenced by rapid remission of clinical signs and survival times extending to the duration of study in most cases. Importantly, several limitations were notable in the design and conduct of these studies. There were a variety of FIP forms and stages of infection among the study cats. In the clinical studies of cats with naturally occurring disease (Pedersen et al., 2018; and Pedersen et al., 2019), cats had different levels of disease progression and associated complications. As a result, the course of treatment and protocols were not uniform among the studies. In addition, the duration of treatment and drug doses were not uniform. Some cats, such as those that relapsed following the original treatment regimen, received extended periods of treatment and/or a higher drug dose. Cats that developed neurological signs of FIP, before or after the initial treatment, were either treated for extended periods or euthanised.

Limitations associated with the experimental model of infection (intraperitoneal inoculation of FIPV as carried out by Kim et al., 2016; and Murphy et al., 2018) include that it could be substantially different from spontaneously occurring natural disease, which typically occurs after faecal-oral exposure of young cats to feline coronavirus. In naturally infected cats, FIP only develops in a minority of cases, subsequent to virus mutations that occur within the host and favour macrophage tropism and systemic spread. The experimental infection causes wet FIP in nearly all subjects and natural extraneous factors cannot affect the progression and presentation of the disease, as well as responses to the treatment (Pedersen et al., 2018). Furthermore, naturally occurring FIP is mostly subclinical for several weeks or months prior to the detection of apparent signs, whereas experimental signs often appear within 2–4 weeks and progress rapidly (Pedersen et al., 2018).

The most critical issue with the design of all four interventional studies was the lack of an independent control group (e.g., placebo-based). All four experiments adopted a before-after approach and assumed the disease would have continuously progressed and eventually led to the death of infected cats if the interventions of interest had not been administered. As in many interventional studies, this assumption might not be consistent with reality, and many confounding factors related to animals and their environment may influence the course of the disease and the potential outcomes of treatment. Pedersen et al. (2018 and 2019), who studied client-owned cats, explain that they did not include separate control groups because of ethical considerations relating to the suggested efficacy of these drugs in previous *in vitro* studies. In addition, it has been shown that nearly all cats with clinical FIP will eventually die within a few weeks to a few months of diagnosis with supportive measures alone (Izes et al., 2020; and Kennedy, 2020). While valid, it is sadly true that none of these explanations can lend extra support to the presented level of evidence generated by these studies.

A limitation of the two laboratory-based experiments (Kim et al., 2016; and Murphy et al., 2018) was the small number of cats enrolled in the studies, which could not represent the broad spectrum of FIP presentations and background variables of affected cats in natural circumstances. There were no explanations regarding the chosen sample size in these studies. As a result of the small sample size and variability in the treatments, no analytical statistics were attempted.

The recommended regimen for GS-441524 was 4 mg/kg, subcutaneously (SC), once daily, for at least 12 weeks, excluding neurological forms of FIP (Pedersen et al., 2019). As for neurological FIP, adequate evidence was not available to support the higher dosage and duration of treatment (compared to non-neurological cases) suggested by Dickinson et al. (2020) in their small case report. The two GC376-based trials also demonstrated the safety and efficacy of the drug in reversing the clinical signs and extending survival times of the majority of FIP subjects (excluding the neurological cases) using a maximum of 15 mg/kg, twice daily, SC for at least 12 weeks (Pedersen et al., 2018). The collective evidence suggests that the potential side-effects of these treatments are mainly limited to transient pain upon injection (with dermal reaction), localised hair loss and/or ulceration of the injection site due to repeated injections. However, retarded juvenile dentition development was reported in four kittens (3–5 months old) associated with long-term treatment with GC376 (Pedersen et al., 2018). 

In conclusion, although all of the reviewed studies suggest that the administration of GC376 or GS-441524 can extend the survival times of cats with FIP, even though relapses and adverse outcomes were reported, the presented evidence is assessed to have moderate strength due to the outlined limitations. Well-designed randomised controlled trials are still required to increase the level of evidence in favor of using these antiviral drugs for clinical cases in the future. However, the ethical dilemmas of conducting double blinded placebo-controlled trials, which by necessity include untreated cats with an invariably fatal disease are recognised.

## Methodology Section

**Search Strategy:** 

Databases searched and dates covered:	CAB Abstracts on EBSCO host: From 1973 to week 43, 2021 PubMed via NCBI: From 1910 to week 43, 2021
Search strategy:	CAB Abstracts: ("feline infectious peritonitis") AND ((nucleoside analog*) OR (GS-441524) OR (Protease inhibitor*) OR (GC376)) PubMed: ("feline infectious peritonitis") AND ((nucleoside analog*) OR (GS-441524) OR (Protease inhibitor*) OR (GC376))
Dates searches performed:	29 Oct 2021

**Exclusion / Inclusion Criteria:** Eligibility criteria were defined based on the relevance of studies to the PICO question in a clinical setting, where adequate details on the treatment protocols were provided to enable reproducibility.

Exclusion:	Unrelated to PICO *In vitro* studies Studies on non-feline species Single case reports
Inclusion:	Studies related to PICO in English *In vivo* studies Peer-reviewed original articles Presented adequate details on the intervention Included more than one cat

**Search Outcome:** 

Database	Number of results	Excluded – In vitro studies	Excluded – Studies on non-feline species	Excluded – Intervention details	Excluded – Unrelated to PICO question	Excluded – Single case report	Total relevant papers
CAB Abstracts	17	2	1	3	5	1	5
PubMed	37	9	2	8	12	1	5
Total relevant papers when duplicates removed							5

## Conflict of Interest

The authors declare no conflicts of interest.

## Disclaimer

Knowledge Summaries are a peer-reviewed article type which aims to answer a clinical question based on the best available current evidence. It does not override the responsibility of the practitioner. Informed decisions should be made by considering such factors as individual clinical expertise and judgement along with patient’s circumstances and owners’ values. Knowledge Summaries are a resource to help inform and any opinions expressed within the Knowledge Summaries are the author’s own and do not necessarily reflect the view of the RCVS Knowledge. Authors are responsible for the accuracy of the content. While the Editor and Publisher believe that all content herein are in accord with current recommendations and practice at the time of publication, they accept no legal responsibility for any errors or omissions, and make no warranty, express or implied, with respect to material contained within. For further information please refer to our Terms of Use.
